# Genome-Scale Phylogeny and Evolutionary Analysis of Ross River Virus Reveals Periodic Sweeps of Lineage Dominance in Western Australia, 1977–2014

**DOI:** 10.1128/JVI.01234-19

**Published:** 2020-01-06

**Authors:** Alice Michie, Vijaykrishna Dhanasekaran, Michael D. A. Lindsay, Peter J. Neville, Jay Nicholson, Andrew Jardine, John S. Mackenzie, David W. Smith, Allison Imrie

**Affiliations:** a School of Biomedical Sciences, University of Western Australia, Perth, Australia; b Department of Microbiology, Biomedicine Discovery Institute, Monash University, Melbourne, Australia; c Environmental Health Hazards, Department of Health, Perth, Western Australia, Australia; d PathWest Laboratory Medicine Western Australia, Perth, Australia; e Faculty of Health Sciences, Curtin University, Bentley, Western Australia, Australia; Cornell University

**Keywords:** *Aedes camptorhynchus*, Western Australia, alphavirus, arbovirus, evolutionary analysis, mosquito, phylogeny

## Abstract

Ross River virus (RRV), an alphavirus of the *Togaviridae* family, is the most medically significant mosquito-borne virus of Australia. Past RRV phylogenetic and evolutionary analyses have been based on partial genome analyses only. Three geographically distinct RRV lineages, the Eastern, the Western, and the supposedly extinct North-Eastern lineage, were classified previously. We sought to expand on past phylogenies through robust genome-scale phylogeny to better understand RRV genetic diversity and evolutionary dynamics. We analyzed 106 RRV complete coding sequences, which included 13 genomes available on NCBI and 94 novel sequences derived for this study, sampled throughout Western Australia (1977–2014) and during the substantial Pacific Islands RRV epidemic (1979–1980). Our final data set comprised isolates sampled over 59 years (1959–2018) from a range of locations. Four distinct genotypes were defined, with the newly described genotype 4 (G4) found to be the contemporary lineage circulating in Western Australia. The prior geographical classification of RRV lineages was not supported by our findings, with evidence of geographical and temporal cocirculation of distinct genetic groups. Bayesian Markov chain Monte Carlo (MCMC) analysis revealed that RRV lineages diverged from a common ancestor approximately 94 years ago, with distinct lineages emerging roughly every 10 years over the past 50 years in periodic bursts of genetic diversity. Our study has enabled a more robust analysis of RRV evolutionary history and resolved greater genetic diversity that had been previously defined by partial E2 gene analysis.

**IMPORTANCE** Ross River virus (RRV) causes the most common mosquito-borne infection in Australia and causes a significant burden of suffering to infected individuals as well as being a large burden to the Australian economy. The genetic diversity of RRV and its evolutionary history have so far only been studied using partial E2 gene analysis with a limited number of isolates. Robust whole-genome analysis has not yet been conducted. This study generated 94 novel near-whole-genome sequences to investigate the evolutionary history of RRV to better understand its genetic diversity through comprehensive whole-genome phylogeny. A better understanding of RRV genetic diversity will enable better diagnostics, surveillance, and potential future vaccine design.

## Abstract (precis)

Ross River virus (RRV) causes the most common mosquito-borne infection in Australia and causes a significant burden of suffering to infected individuals as well as being a large burden to the Australian economy. The genetic diversity of RRV and its evolutionary history have so far only been studied using partial E2 gene analysis with a limited number of isolates. Robust whole-genome analysis has not yet been conducted. This study generated 94 novel near-whole-genome sequences to investigate the evolutionary history of RRV to better understand its genetic diversity through comprehensive whole-genome phylogeny. A better understanding of RRV genetic diversity will enable better diagnostics, surveillance, and potential future vaccine design.

## INTRODUCTION

Ross River virus (RRV) is the most common cause of arbovirus-induced disease in Australia, with approximately 5,000 clinical cases reported annually. Infection with RRV is associated with malaise, myalgia, rash, and potentially persistent and debilitating joint symptoms (1). People living in the tropical and subtropical regions of northern Queensland, Western Australia, and the Northern Territory are at highest risk of infection (1, 2). Disease notifications demonstrate seasonality, based on ideal climatic and environmental conditions that influence vector and reservoir populations, generally during the warmer months (January to April) (3).

Regarded as both a vector and vertebrate host generalist, RRV transmission is maintained in a sylvatic cycle between diverse mosquito vector species and vertebrate amplifying hosts (reviewed in references 4 and 5). Based on serological surveys and infection studies, macropods (kangaroos and wallabies) are considered to be the most significant vertebrate hosts of RRV in Australia (6). Humans are often incidental dead-end hosts in this enzootic cycle, with a typical infection resulting in low-magnitude short-lasting viremia that is insufficient for ongoing transmission (7). A large virgin-soil RRV epidemic occurred in multiple Pacific Island countries and territories (PICTs) in 1979–1980, infecting roughly 500,000 individuals (8). In the absence of macropod hosts in the PICTs, an epidemic human-mosquito-human transmission cycle has been suggested for this outbreak, possibly initiated in Fiji by a viremic traveler from Eastern Australia (9, 10). The PICTs outbreak highlights the potential for spatial expansion and large epidemics of RRV in naive human populations where a competent vector is present (11).

RRV belongs to the *Alphavirus* genus, within the *Togaviridae* family. The single-stranded, positive-sensed RNA genomes are approximately 11.8 kb and encode both nonstructural (nsP1 to -4) and structural (C, E3, E2, 6K, and E1) genes through two separate open reading frames (ORFs) (12–14). Structural and nonstructural genes are initially translated as separate polyproteins, which are subsequently autocatalytically cleaved to produce the individual protein products. The genome is flanked by 5′ and 3′ untranslated regions (UTRs) (15). Previous RRV phylogenetic studies based on partial E2 analysis of a limited number of RRV isolates identified three distinct and divergent RRV genotypes (G1 to G3), described as displaying strong geographical structure within “North-Eastern” (G1), “Western” (G2), and “Eastern” (G3) Australian lineages (10, 11). It has been proposed that G3 viruses, which were first detected during the PICTs epidemic, replaced both G1 and G2 viruses following the resolution of the outbreak and are both currently extinct or in low circulation (11, 16).

Western Australia (WA) is the largest Australian state, making up a total land mass of 2.5 million km^2^, an area considerably larger than most countries. The northern and western coasts of WA meet the Indian Ocean, while the southern coast is bounded by the Southern Ocean. Despite being geographically vast, only approximately 10% of the Australian population live in WA and >80% of Western Australians reside in the southwest corner, where the state’s capital, Perth, is located (17, 18). RRV transmission and disease cases can occur in any of the state’s three major climatic regions: the tropical/subtropical north, the temperate south, and the central arid regions (1). Activity of medically significant arboviruses, including RRV, has been monitored in northern WA since the 1970s through an annual mosquito trapping program and intermittent opportunistic sampling (19, 20). More regular mosquito sampling is nonviable due to the logistical barriers of accessing remote areas of the Kimberley and Pilbara (21). A routine surveillance program based in the more heavily populated southwestern regions of WA has been in place since 1987, with regular trapping of mosquitoes in high RRV-risk areas along the coast (19, 22). Viruses isolated from trapped mosquitoes are identified with specific monoclonal antibodies in a fixed-cell enzyme-linked immunosorbent assay (ELISA) and, more recently, reverse transcriptase PCR (RT-PCR) (19, 23). To date, there has yet to be a genome-scale phylogenetic study conducted on RRV in Australia, with only 13 unique whole-genome sequences published and available on NCBI as of July 2019, which includes a single isolate from Western Australia (DC5692, accession number HM234643).

To infer the spatiotemporal evolution of RRV in Australia and to better define its evolutionary dynamics and genetic diversity, we conducted a genome-scale phylogenetic and evolutionary analysis of mosquito- and human-derived RRV isolates from a range of Western Australian locations between 1977 and 2014. Sequences were analyzed with publicly available whole-genomes previously deposited in GenBank. Whole-genome analysis will provide more robust and comprehensive estimates of Ross River virus evolutionary rates and assessment of evolutionary history and relationships between distinct isolates.

## RESULTS

### Whole-genome sequencing of Ross River virus isolates.

**FIG 1:**
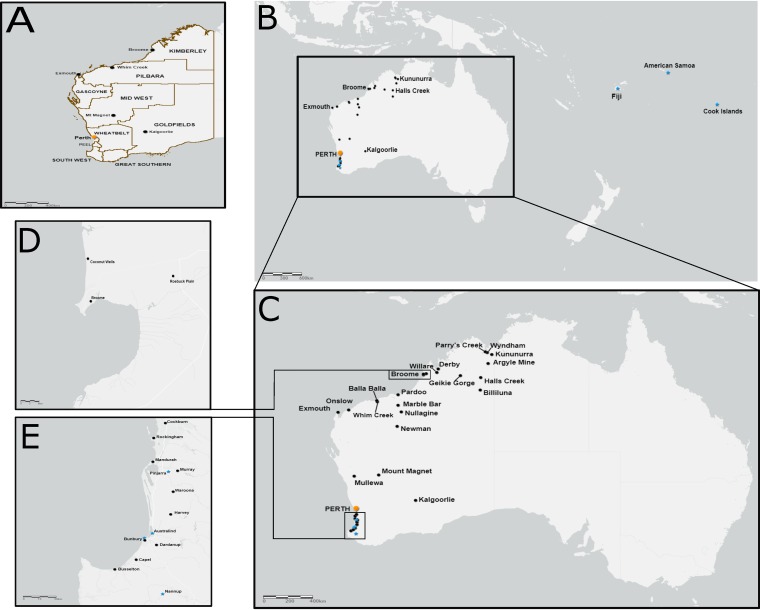
Sampling locations of RRV isolates sequenced in this study. (A) Map indicates the boundaries of the major Western Australian regions. (B to E) Black circles indicate where mosquito-derived isolates were sampled, while blue stars indicate locations of human-derived RRV isolate sampling. All maps were generated using Arc GIS (ESRI).

The complete coding genomes of 94 RRV isolates were successfully sequenced in this study (see Table S1 in the supplemental material). For all isolates sequenced, only partial 5′ and 3′ UTR sequences were resolved. Seventy-seven mosquito-derived isolates were selected to represent a wide spatial and temporal range (1977–2014) within Western Australia (Fig. 1). Four human-derived RRV isolates, each sampled in the south of Western Australia (1989–1992), were included as well as 13 clinical isolates collected from residents of Fiji, American Samoa, and the Cook Islands during the PICTs RRV disease epidemic (1979–1980). In addition, all publicly available RRV genomes were downloaded and included in all analyses with the exception of NB5092 (New South Wales, 1969, accession number M20162), which was excluded due to high nucleotide ambiguity. Our final data set consisted of the complete coding regions of 106 RRV genomes sampled over a 59-year time period from 1959 to 2018.

The average pairwise nucleotide identities of all genome regions, as well as additional gene properties, are presented in Table 1. Single nucleotide polymorphisms (SNPs) were observed throughout the genome. The nsP3 gene was the most variable of the nine RRV genes studied in this data set, with an average pairwise (PW) nucleotide identity of 95.2%. The nonstructural genes, nsP1 and nsP2, and the structural genes, E1 and E2, were the most conserved genome regions studied (average PW identity, 98.0% to 98.4%).

**TABLE 1:** The length (excluding gaps), pairwise nucleotide identities, and base frequencies of individual genes within the 106 taxa Ross River virus data set

Gene region	Gene region length including gaps (nt/aa)	Avg pairwise identity (nt/aa) (%)	Base frequencies (A, C, G, T) (%)
nsP1	1,602/534	98.4/99.0	(28.8, 24.7, 26.7, 19.9)
nsP2	2,394/798	98.3/99.5	(28.1, 24.4, 26.4, 21.2)
nsP3	1,650/550	95.2/96.5	(24.0, 26.3, 29.0, 20.7)
nsP4	1,833/661	97.9/99.2	(28.5, 23.9, 26.5, 21.1)
C	810/270	98.5/99.1	(33.4, 24.9, 26.7, 15.1)
6K	180/60	98.8/98.1	(19.8, 25.4, 25.5, 29.3)
E1	1,314/438	98.1/99.6	(26.3, 26.6, 26.4, 20.7)
E2	1,266/422	98.0/99.3	(25.2, 28.2, 26.9, 19.8)
E3	192/64	97.2/99.5	(24.8, 32.2, 22.3, 20.6)

### Phylogenetic relationships and spatial movement of RRV.

**FIG 2:**
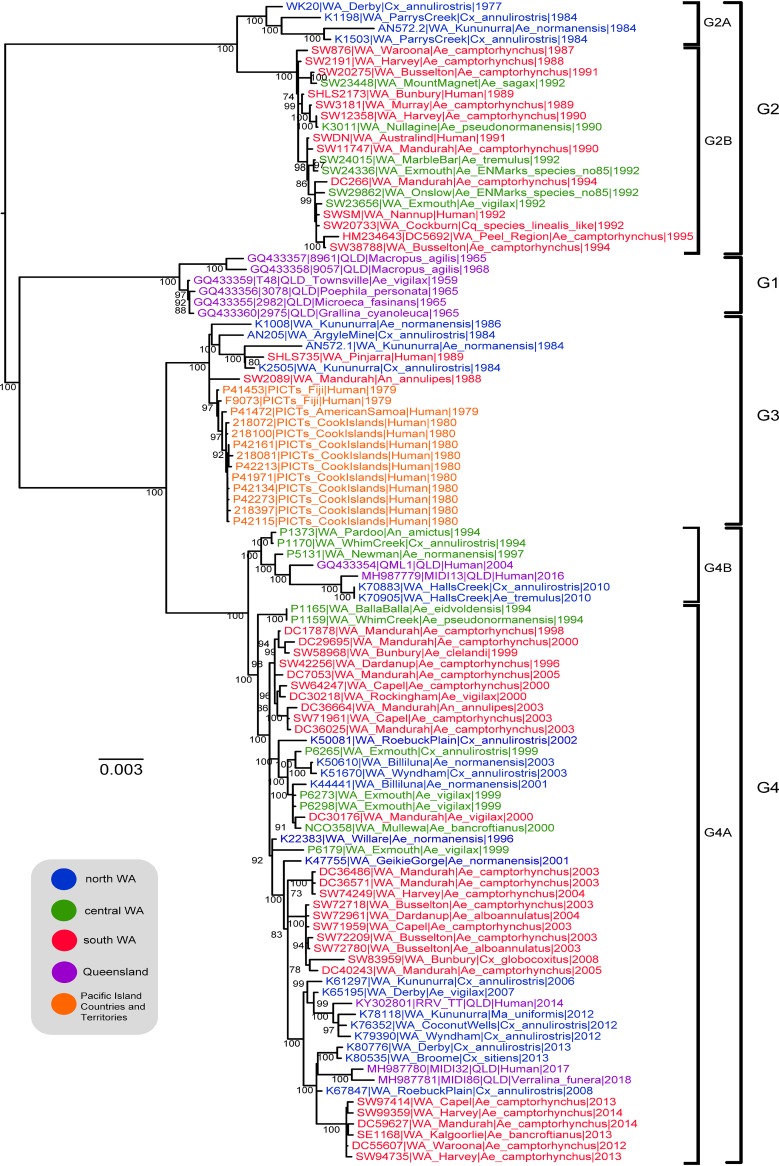
Maximum likelihood phylogeny (RAxML) reconstruction of 106 RRV whole-genome sequences. Virus nomenclature includes the strain name, location of collection, species of origin, and the year of sampling. GenBank accession numbers are provided for sequences derived from NCBI. Taxa are colored based on their geographical origin. Bootstrap support values >70% are presented above nodes.

Phylogenetic analysis of the complete RRV coding sequence data set revealed the historical circulation of two of the three previously described RRV genotypes (G2 and G3) within WA as well as a newly classified fourth genotype (G4) that was most closely related to G3 (Fig. 2). No isolates belonging to G1, which contains the RRV prototype, T48, were detected within WA during our study period. Nonparametric bootstrapping showed strong support (100% of 1,000 bootstrap replicates) for each of the four genotypes. The majority of isolates sampled in this data set (58 of 106) and all isolates collected in WA since 1996 belonged to the G4 genotype, indicating that it is the contemporary and dominant genotype in circulation in Western Australia. Recently sampled isolates from QLD (2016–2018) were also classified as G4, indicating that this group may also be in current circulation in the east of Australia. Prior to the first detection of G4 viruses in WA in 1994, G2 viruses were the predominant group in this data set (1977–1995, *n* = 22) detected throughout Western Australia. A small number of G3 isolates (*n* = 6) were detected during the 1980s, but this represented a large proportion (6/13 [46%]) of the isolates sampled within WA during that period.

G2 viruses (*n* = 22) formed two distinct and well-supported sublineages, separated spatially and temporally within WA. G2 viruses sampled 1977–1984 were collected in the Kimberley region of northern WA (sublineage G2A), whereas isolates sampled subsequent to this period, between 1987 and 1995, were composed exclusively of samples collected from southern and central WA (sublineage G2B). Three human-derived Western Australian RRV isolates, collected 1989–1992, clustered with the southern G2B sublineage. It may appear as though the G2A lineage in the north was the source of the G2B lineage in the south, based on their sampling dates. However, it must be noted that virus sampling in the north predates sampling in the south of WA, and there are no data to indicate what viruses were in circulation in the south prior to 1987. Interestingly, the northern G2A lineage demonstrated greater genetic diversity than the southern G2B lineage.

The detection of multiple isolates from the G2 and G3 genotypes within Western Australia, which were previously classified as the Eastern and Western Australian lineages, respectively, calls into question the supposedly strong nationwide geographical structure of RRV genetic groups. Further evidence of this lack of strong nationwide geographical structure is evident when a maximum likelihood phylogeny was constructed with our whole-genome sequencing (WGS) data set and published partial E2 gene sequences (see Fig. S1). Notably, G3, previously designated the Eastern lineage, contained isolates sampled from QLD, WA, Tasmania, New South Wales, Victoria, and the Northern Territory, all within a 5-year period (1983–1988).

Interestingly, G2 and G3 viruses appear to have cocirculated in northern WA in the early 1980s and again in the south in the late 1980s. On both occasions, G2 was likely dominant in the local region prior to cocirculation and remained so after cocirculation was observed. This is the first observation of spatial and temporal cocirculation of two distinct RRV genetic groups. Two separate and distinct viruses were derived from isolate AN572, sampled from Kununurra in northern WA in 1984, one of which grouped with G2 (AN572.2) and the other with G3 (AN572.1), exemplifying the spatial and temporal cocirculation of these two genetic groups.

All isolates originating from the Pacific Islands epidemic (1979–1980) grouped within G3 and shared a high degree of nucleotide identity (99.6% to 100% average pairwise identity). Among the six Western Australian-derived G3 isolates (1984–1989), four were derived from north WA and two were sampled in the south of Perth. Previous studies concluded that the Pacific Islands epidemic was initiated by the introduction of RRV into the Pacific by an infected Eastern Australian traveler, as Pacific RRV isolates grouped with the Eastern RRV lineage (10). Our data, based on analysis of isolates sampled from throughout WA soon after the epidemic interval, do not support the previous geographical classification of RRV genetic groups and also identified closely related viruses within WA close to the resolution of the PICTs epidemic. Analysis of a larger number of isolates from throughout Australia from this period may resolve the origins of the Pacific Islands epidemics.

Genomic analysis revealed the expansion of genetic diversity among the emergent G4 viruses since the early 1990s, with two major sublineages characterized thus far. The majority of G4 isolates (51 of 58 isolates, 1994–2014) belonged to a distinct sublineage (G4A) which included several recent human-derived isolates from QLD. The second major sublineage (G4B) contains several mosquito-derived isolates from north and central WA (1994–2010) as well as two human-derived isolates sampled in QLD in 2004 (QML1) and 2016 (MIDI13). Interestingly, the earliest isolates of both distinct sublineages were isolated from mosquitoes collected on the same day and location in 1994 at Whim Creek in the Pilbara region. The mechanism by which G4 seemingly became the dominant lineage of RRV in circulation is yet to be elucidated.

### Evolutionary history of RRV genetic groups.

**FIG 3:**
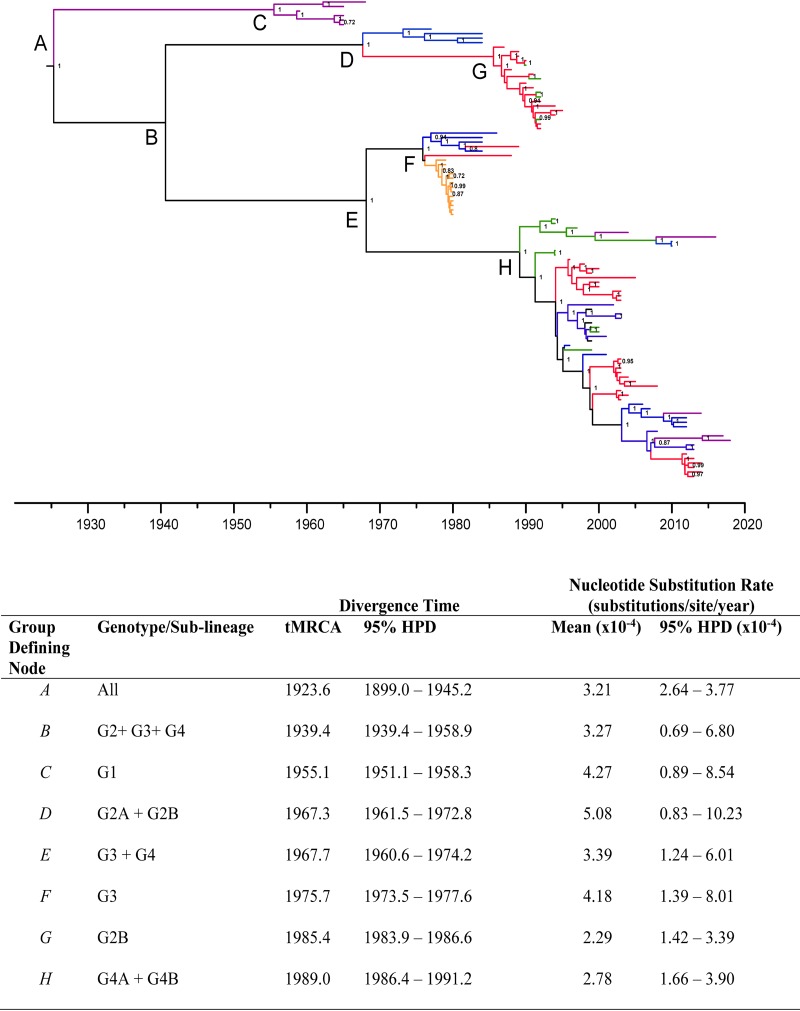
Maximum clade credibility tree (MCC) of 106 dated Ross River virus whole-genome sequences, estimated under an uncorrelated log normal (UCLN) molecular clock, assuming a GTR + G + I nucleotide substitution model. Clades are colored for the geographical origin of the taxa. Posterior probability values of >0.70 are shown above branches. Nodes defining major genetic groups are named A to H and are referenced in the lower table. Calendar years are shown on the *x* axis. The table presents the divergence time (time to most recent common ancestor [tMRCA]) of distinct nodes (A to H) and the nucleotide substitution rates, with statistical error reported as the 95% highest probability density (95% HPD).

To infer the time scale of emergence of RRV genotypes, we estimated the time to most recent common ancestor (tMRCA) and evolutionary rates of each defined genetic group based on a maximum clade credibility (MCC) phylogeny (Fig. 3) under an uncorrelated log normal (UCLN) relaxed molecular clock (24). The tMRCA of the root of the RRV phylogeny was estimated as 1923 (95% highest probable density [HPD], January 1899 to February 1945), or approximately 94 years ago. The mean divergence times of each RRV genotype occurred over the past 50 years, each separated by approximately 10 years (Fig. 3). Incidentally, the divergence time of the G3 and G4 groups in approximately 1967 (95% HPD, 1960.6 to 1974.2) coincided with the tMRCA of G2 (mean, 1967.3; 95% HPD, 1961.5 to 1972.8).

Our estimate of the overall tMRCA was somewhat older than estimated previously in an analysis based on a partial E2 gene (<250 bp) data set with 62 dated taxa (mean, 1949; 95% HPD, 1925–1959), indicating that we have characterized a greater diversity of RRV using near-whole-genome sequences and a larger data set (11). It should be noted that the partial E2 data set lacked a strong temporal signal as demonstrated by root-to-tip regression analysis (*R*^2^ = 0.429); thus, estimates of tMRCA and nucleotide substitution rates derived from this data set may not be reliable.

### Ross River virus evolutionary rates and selection pressure.

The mean nucleotide substitution rate (substitutions/site/year) estimated using the whole-genome data set of RRV under a relaxed UCLN molecular clock was 3.21 × 10^−4^ substitutions/site/year (95% HPD, 2.64 × 10^−4^ to 3.77 × 10^−4^) (Fig. 3). These estimates are consistent with previous estimations of alphavirus substitution rates based on whole-genome sequences and the observations of this study (see below) that RRV, while being a low-fidelity RNA virus, evolves under a high degree of negative selection pressure given that it must replicate in two very distinct host systems, the mosquito and the mammalian host (25, 26).

Selection pressure analysis revealed that the entire RRV genome is under purifying selection, with many sites under significant negative selection pressure within each ORF (between 106 and 830 individual sites). This is consistent with the widely supported “trade-off” hypothesis for mosquito-borne alphaviruses, that is, that the alternation of replication between two distinct host systems (vertebrate and invertebrate) limits the evolution of arboviruses, as enhanced fitness in one host may be detrimental to replication in the alternate host (27).

In contrast to previous investigations of RRV selection pressure, five individual codon sites were found to have significant evidence of positive selection pressure by at least two methods utilized in the analysis (Table 2), within the nonstructural (nsP1, two sites; nsP3, one site) and structural (E1, one site; E3, one site) polyproteins (11). Notably, the site under apparent positive selection pressure within nsP3 was found to be within the N terminus of the hypervariable domain (HVD). The I248T substitution within the nsP1 gene was the most highly represented site under positive selection, was observed in 40 individual isolates from all four genotypes, and was supported as a site under positive selection in all four selection analysis methods implemented.

**TABLE 2:** Codon sites with significant evidence of positive selection pressure

Codon site^*a*^	Selection detection method^*b*^				Amino acid substitution and genome location	Sequences with derived amino acid state
	FEL	SLAC	MEME	FUBAR		
Nonstructural polyproteins						
248	**0.001**	**0.003**	**0.0027**	**0.999**	I248T, nsP1	G1: 9057, 8961
						G2: SW12358, K3011, WK20, AN572.2
						G3: AN572.1, AN205, SW2089, P42134, P41472, P41453, P42161, P42273, P42115, P41971
						G4: P5131, QML1, SW99359, SE1168, DC59627, SW94735, MIDI13, SW94735, SW97414, DC55607, RRV_TT, K79390, K78118, K76352, K80776, K80535, K67847, K65195, K61297, DC36486, SW72780, SW72209, SW64247, DC29695
441	0.085	0.158	**0.042**	**0.982**	K441E, nsP1	G2: WK20
						G3: AN205, P41472, F9073, 218100, 218072
						G4: MIDI86
1165	0.080	1.00	**0.05**	**0.965**	A333T, nsP3	G3: AN205
						G4: DC40243, K80535, K80776
					A333V, nsP3	G4: DC30218, SW64247, DC36664, SW71961, DC36025
Structural polyproteins						
329	**0.045**	0.196	0.06	**0.977**	G59R, E3	G1: 2982, T48, 3078, 2975, 8961, 9057
						G3: K2505, SHLS735, AN72.1
					G59E, E3	G4: DC40243, SW71959, SW72718, SW72780, SW72961, SW72209, SW83959
929	**0.039**	0.208	0.06	**0.974**	V113I, E1	All G3 (except AN572.1), All G4 (except K50610, K51670, DC55607, SE1168, SW97414, DC59627, SW94735, SW99359, K67847, K80535, K80776, MIDI32, MIDI86)

a Refers to amino acid positions within either the nonstructural or structural polyprotein.

b FEL, fixed-effect likelihood; SLAC, single-likelihood ancestor counting; MEME, mixed-effects model of evolution; FUBAR, fast, unconstrained Bayesian approximation. Significant selection sites confirmed by at least two of these methods as well as the isolates that contain the corresponding amino acid substitution are in boldface font.

### Phylodynamics of Ross River viruses.

Collectively, the phylogenetic structure and tMRCA estimates of the RRV genotypes suggest that long-term RRV evolution is characterized by periodic bursts in genetic diversity, likely signifying the emergence of fit variants. To investigate changes in demographic patterns of RRV over time, we estimated the changes in relative genetic diversity using a flexible Bayesian coalescent skyline model that allows for the characterization of temporal changes in demographic history. The Bayesian skyline plot estimates changes in effective population size through time under the assumption of the absence of selection pressure; however, since RRVs are known to undergo selection pressure (also see the selection analysis), we infer our results as changes in relative genetic diversity to be consistent with previous studies (11).

**FIG 4:**
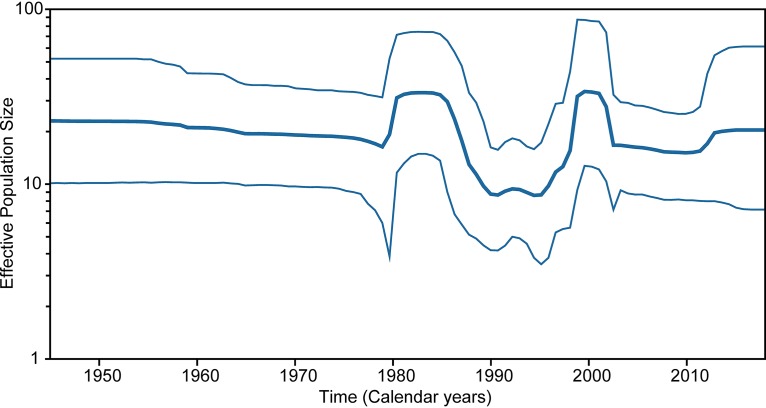
Bayesian skyline plot demonstrating fluctuations in relative RRV effective population size (*y* axis) through time, in calendar years (*x* axis). The center line demonstrates the mean estimate of effective population size, with the upper and lower lines showing statistical error as the 95% highest probability density (95% HPD).

The Bayesian skyline analysis showed substantial changes in RRV relative genetic diversity, with peaks in diversity during the early 1980s and during 2000 (Fig. 4). The lower levels of genetic diversity observed during the remaining periods were similar to those estimated using the E2 genes previously, although these estimates were lower than those estimated for other globally prevalent arboviruses such as dengue virus and chikungunya virus (CHIKV) (11, 28, 29). Considerable increases in virus effective population size was detected during early to mid-1980s, coinciding with the PICTs epidemic, and during late 1990/early 2000, likely signifying the rapid expansion of G4 during this time.

### Amino acid variability analysis.

Complete coding sequence alignment revealed conservative and nonconservative amino acid substitutions in each gene of the RRV genome. Each distinct genetic group G1 to G4 was defined by unique amino acid residues (see Table S2). It should be noted that even single amino acid substitutions at critical sites of the genome have influenced the epidemiology of significant alphaviruses in the past. The E1-A226V transition in the chikungunya virus (CHIKV) genome enhanced transmission of this variant by Aedes albopictus, a globally dispersed vector, allowing for the spread of CHIKV to new regions following the 2006 Reunion Island CHIKV epidemic (30).

Most amino acid transitions in our data set were observed within the C-terminal region of the nsP3 gene, otherwise known as the hypervariable domain (HVD) due to the high variability in nucleotide sequence and length, even between strains of the same alphavirus species (31). The HVD has been observed in alphaviruses such as Semliki Forest virus (SFV) and CHIKV to be able to withstand large nucleotide insertions and deletions, with minimal impact on viral replication efficiency (16). While this domain is largely nonconserved among the alphaviruses, the HVD contains several conserved elements, such as the proline-rich (P*P*PR) domains and duplicated FGDF-like motifs that are conserved among all Old World alphaviruses and which interact with host proteins to influence viral replication (31, 32). Four repeat P*P*PR motifs have been observed within the RRV nsP3 (16).

Nucleotide deletions of various lengths (3 nucleotides [nt] to 135 nt, 1 to 45 amino acids [aa]), within the HVD were observed for 24 isolates belonging to G2 to G4 in our data set (Table 3). Interestingly, most observed nucleotide deletions, with the exception of those occurring in two isolates, K3011 and DC5692, resulted in the loss or partial loss of either the second or third RRV HVD P*P*PR motif. While the supposedly well-conserved proline motifs of the HVD were removed in several isolates studied, all RRV isolates in our study maintained both conserved FGDF-like motifs.

**TABLE 3:** RRV isolates with observed deletions within the hypervariable domain of the nsP3 gene^*a*^

Isolate name	Virus genotype	Genomic location of deletion (nt position)	P*P*PR motif affected	Size of deletion (nt)
DC5692	2	5414–5416	None	3
SW2191	2	5378–5416	2	39
K1198	2	5378–5416	2	39
K3011	2	5309–5383	None	75
SW24015	2	5336–5416	2	81
SW29862	2	5338–5418	2	81
SW2089	3	5434–5446	3	33
P42134	3	5380–5415	2	36
SW42256	4	5380–5415	2	36
SW83959	4	5380–5415	2	36
K50081	4	5380–5415	2	36
SW94735	4	5379–5414	2	36
DC7053	4	5377–5415	2	39
DC29695	4	5377–5415	2	39
DC36486	4	5377–5415	2	39
SW72209	4	5375–5413	2	39
K65195	4	5356–5415	2	60
P5131	4	5356–5415	2	60
SW72780	4	5356–5415	2	60
DC55607	4	5338–5415	2	78
K79390	4	5338–5415	2	78
SW97414	4	5338–5415	2	78
SW72718	4	5312–5413	2	102
SW74249	4	5281–5415	2	135

a For most isolates, these deletion events resulted in the loss or partial loss of one of the four conserved RRV proline (P*P*PR) motifs within nsP3. The identity of the affected proline motif (numbered 1 to 4) is presented for each deletion. The genotype of the isolate and the genomic location and size of the deletion events are also presented.

**FIG 5:**
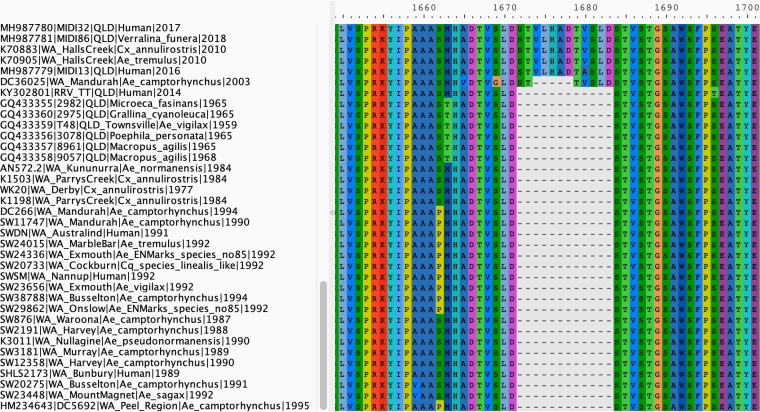
MAFFT alignment of the Ross River virus data set revealed a 12-amino-acid insertion within the hypervariable region of the nsP3 gene, which was unique and characteristic of G3 and G4 isolates. Two isolates, DC36025 and RRV_TT, had a 5-amino-acid and 12-amino-acid deletion within this insertion region, respectively. No G1 or G2 isolates in our study contained this 36-nucleotide insertion.

Another notable observation was that of a 12-amino-acid insertion (STVLHADTVSLD) within nsP3, upstream of those deletion events noted above (Fig. 5). This insertion was observed in all G3 and G4 viruses and not in the other genotypes. The earliest isolate in this data set that contained this insertion was G3 isolate F9073, collected in Fiji in 1979 at the beginning of the PICTs epidemic. Nucleotide deletions were observed within this apparently fixed insertion region, in isolates DC36025 and RRV_TT (accession KY302801). The 15-nt deletion within DC36025 resulted in the loss of the V-L-H-A-D portion of the insertion sequence. The 36-nt deletion observed within RRV_TT occurred from the second nucleotide within the insertion region, resulting in the complete loss of the 12-aa sequence, and was the only isolate of G3 or G4 in our data set to lack this insertion. The events leading to the fixation of this 12-aa element are yet to be resolved.

An arginine residue (CGA) in place of an opal termination stop codon (UGA) at the C terminus of the nsP3 gene was observed in isolate K3011 (G2), isolated from a pool of Aedes pseudonormanensis mosquitoes sampled in the Pilbara town of Nullagine in 1990. Such opal stop codon-to-arginine transitions have been observed in several strains of other alphaviruses, including O’nyong-nyong virus (ONNV) (33) and CHIKV, and this is the first documented observation of such a transition in RRV. Introduction of an arginine codon into a CHIKV isolate significantly reduced joint morbidities in infected mice compared to that in mice infected with the wild type (34). Virus replication did not appear to be affected. ONNV isolates that contained the opal stop codon all demonstrated the opal-to-arginine residue change by the fifth passage on Vero cells, suggesting that variants of ONNV containing both residues exist as a quasispecies and have different abilities to replicate in different hosts (31, 35). In the present study, an opal stop codon-to-cysteine (TGT) transition was observed in the nsP3 gene of P42213 (G3), an isolate from the Cook Islands sampled at the conclusion of the PICTs epidemic in 1980. Such a transition has been described in an isolate of Sindbis virus (SINV; S.A.AR86) and was associated with neurovirulence in mice, in comparison with a similar avirulent SINV strain, Girdwood, which retained the opal codon (36, 37).

The potential biological implications of these observations in relation to RRV pathogenicity, replicative fitness, and transmission dynamics are not yet known and should be explored.

## DISCUSSION

In this study, we sought to better understand the evolutionary history and genomic diversity of Ross River virus (RRV) and to expand the current phylogeny which, until now, had been based on a small portion of the E2 gene. We generated the most informative RRV data set to date with 94 new and 13 previously published near-whole-genome RRV sequences. This data set has enabled a more thorough and robust analysis of RRV phylogeny and evolutionary history and has allowed potentially significant observations within genome regions that had not been extensively studied previously. We identified four distinct genotypes (G1 to G4) of RRV, including the newly described G4, that have circulated in Australia during our 59-year study period.

Among the most significant findings was the identification of a long-term pattern of RRV evolution, whereby RRV genotypes emerge periodically, roughly, every decade. Emergent lineages were observed to demonstrate extensive geographical movement in a relatively short time frame. Sweeps in genotype dominance were observed in our investigation, whereby previously dominating genotypes were replaced by newly emergent variants. Within Western Australia, for example, G2 appeared to have been the dominant lineage in circulation between 1977 and 1995. G4 viruses were first detected in WA in 1994 and appeared to quickly become the dominating lineage throughout the state, with no G2 or G3 isolates detected in WA, or Australia as a whole, since 1995. The potential biological and ecological drivers of these apparent sweeps of genotype dominance within Western Australia are yet to be elucidated.

Virus isolates sequenced in our analysis were derived from a wide range of mosquito vector species. RRV is a vector generalist and has been isolated from over 40 unique species of mosquito across Australia (6). The diverse climates, landscapes, and weather patterns among the southern, central, and northern regions of Western Australia result in different mosquito breeding conditions and species distributions. For example, in the tropical northern regions of WA, where freshwater breeding sites associated with floodplains are plentiful, Culex annulirostris is generally the dominant vector species. In the south, specifically along the Swan Coastal plan, saltwater marsh mosquitoes such as Aedes vigilax and Aedes camptorhynchus dominate (38). In general, a greater diversity of mosquito species is observed in WA’s north than in the south. The genetic diversity among the distinct RRV genotypes characterized in this study may have some impact on vector competence, and it is possible that mosquito ecology may potentially influence the distribution and evolution of RRV in WA. Our preliminary analysis has not identified any unique or characteristic genetic features that are shared among viruses derived from specific mosquito species, but this will be investigated further in the future. The northern G2A lineage was observed in our study to be more genetically diverse than the southern G2B lineage. Genetic diversity of G2A may be driven by the greater diversity of mosquito species present in the north or potentially by the likely greater transmission that occurs among potentially larger populations of amplifying hosts in the state’s rural north. Long-term endemicity of G2A in the state’s north may also explain the genetic variability between isolates. The near identical clusters of sequences sampled in the south may indicate continual seeding of virus from other locations, possibly, the north of WA or interstate.

Western Australian viruses analyzed in this study were sampled during routine mosquito-based surveillance conducted by the WA Department of Health and the University of Western Australia. The surveillance program was initiated in the southwest region, where the majority of the WA population resides, in 1987, and the earliest samples from the south in our data set date to this time. Prior to this, study isolates derived from northern WA regions in 1977–1986 were collected as part of an independent research project (39). Mosquito-based surveillance activities in the central, remote, and arid regions of WA are only usually conducted opportunistically in response to extreme weather events and are not conducted as frequently as in the more populous regions of the south with larger at-risk populations. Central WA isolates are more limited for this reason. The number of available virus isolates greatly increased from the mid-1990s onwards, when the Western Australian mosquito trapping program had become well established. For these reasons, the densities of samples collected per year and per location within Western Australia were not uniform. All attempts were made to maximize the spatiotemporal range of isolates through the selection of samples to represent this wide range, where possible.

The previous classification of RRV genetic groups into geographically distinct lineages, that is, the North-Eastern (G1), Western (G2), and Eastern (G3) Australian lineages, is not supported by the findings of this study, and we propose that this nomenclature is no longer valid. The Eastern lineage was composed of isolates sampled Australia wide. The contemporary G4 lineage was also found to be geographically dispersed, with isolates sampled in the west and east of Australia. While isolates grouping with the Western lineage were only detected in Western Australia and the Northern Territory, it cannot be said definitively that this genotype is exclusive to the west, as sequence data derived from the eastern states is limited in comparison to our western data set. No isolates of the North-Eastern lineage were sampled in WA during our study period, possibly as our sampling within Western Australia was limited during the time G1 was likely in higher circulation. It is plausible that lineages of RRV were once more geographically structured and only became dispersed with the more recently emergent G3 and G4 genotypes with the advent of increased travel or due to an undefined fitness advantage that supported greater dispersal. A likely possibility is that G1 and G2 were erroneously regarded as geographically distinct due to the lack of representative sampling from a broad range of locations through time in earlier studies.

All viruses within G3 and G4 contained a unique and characteristic 12-amino-acid insertion within the nsP3 region. It has been suggested that this insertion, first observed in G3 isolates derived from the Pacific Islands epidemic, conferred a fitness advantage to RRV that resulted in increased case reporting in Australia and replacement of the G1 and G2 lineages (16). Increased RRV case burden is more likely the result of RRV becoming a notifiable disease in Australia around the time of resolution of the Pacific Islands epidemic. Furthermore, in the present study, we show that G2 viruses circulated in Australia until at least 1995, 15 years following the first detection of G3. Our temporal analysis suggests that the G3/G4 group, and likely the characteristic 12-amino-acid insertion, emerged in approximately 1968 (95% HPD, 1961–1974), 11 years prior to the PICTs epidemic, and so it is unlikely that the insertion itself bears any importance in epidemic fitness. The significance of this apparently fixed 36-nucleotide insertion in terms of RRV fitness and virulence will be explored further.

Replacement of the opal stop codon by an arginine and cysteine residue, as was seen in isolates K3011 and P42214, respectively, has been described for other alphaviruses, including CHIKV and SINV (34, 36), but this is the first time these mutations have been identified in the RRV genome. In addition, the loss of otherwise well-conserved proline-rich domains within the hypervariable domain (HVD) and the nucleotide deletions of various lengths that were observed in multiple isolates may have significant implications in terms of RRV replicative fitness, virulence, vector competence, and human pathogenesis that will need to be investigated. Only partial 5′ and 3′ UTR sequences were resolved for all isolates included in our study. The UTRs of alphaviruses play roles in the regulation of viral gene expression and replication and can significantly influence the host range, vector competence, and pathogenesis of alphaviruses (reviewed in reference 15). Complete sequencing of these regions within the RRV isolates will be pursued in the near future.

In comparison to the phylogeny and evolutionary rates inferred from analysis of the partial E2 (250 bp) gene sequence (11), a greater diversity of RRV was resolved. The partial E2 phylogeny constructed using the isolates sequenced for this study was found to be insufficient to discriminate between the G3 and G4 genotypes, with poor bootstrapping support for most clade-defining nodes. The nucleotide substitution rates and divergence times of lineage-defining nodes estimated with our WGS data set were slower and older than the estimates of the previous partial E2 phylogeny. These previous estimates were inconsistent with estimates derived from other studies of alphavirus evolutionary rates (26).

Our RRV data set was estimated to have diverged from an ancestral strain roughly 94 years ago. Timescales have been estimated for other alphaviruses, including SINV (40), CHIKV (28), Eastern equine encephalitis virus (EEEV) (41), and Venezuelan equine encephalitis virus (42). Making meaningful comparisons of these timescales is currently difficult given the various evolutionary models and data sets utilized in these analyses. It would be informative to compare the timescales of these viruses in the future, when equivalent data sets can be obtained with further whole-genome sequencing studies.

Prior to our investigation of 106 RRV whole genomes, analysis of RRV selection pressure had been restricted to 8 genome sequences and 62 partial E2 gene sequences (11). Consistent with previous reports for vector-borne RNA viruses, both RRV ORFs were observed to be under significant negative selection pressure. Additionally, several codons within the nonstructural (nsP1 248, nsP1 441, and nsP3 333) and structural (E3 5 and E1 113) polyproteins were found to be under significant positive selection pressure. The internal nonstructural proteins of vector-borne RNA viruses, such as RRV, are not expected to differ greatly in selection pressure compared to that of non-vector borne RNA viruses, as these proteins do not interact with host proteins and therefore are not influenced by host alternation (43). We identified sites under diversifying selection within the structural genes, and this has not been observed on many occasions in vector-borne RNA viruses. One site under positive selection was observed in the structural E gene (E 332) of Murray Valley encephalitis virus that likely played a role in immune evasion, conferring a fitness advantage to the dominant lineage (44). The specific structural genome sites under positive pressure that were observed in our data set, within E3 and E1, have not been extensively studied; therefore, the exact consequences of mutations at these sites are difficult to predict. It is of note, however, that substitutions at these sites were mainly observed within isolates of G3 and G4 and may confer a fitness advantage.

Further whole-genome sequencing of isolates sampled from throughout Australia, covering a wide temporal and spatial range, may provide greater certainty to our evolutionary and selection pressure analysis and, in particular, our estimates of divergence times of distinct genetic groups. Continued surveillance and sequencing of RRV isolates, from a wide spatiotemporal range, should be undertaken to monitor the ongoing evolution of RRV into the future.

## MATERIALS AND METHODS

### Virus isolates and RNA extraction.

The virus isolates included in this study are listed and described in Table S1 in the supplemental material. The protocols for the collection, pooling, and processing of mosquito samples to derive and identify virus have been described elsewhere (20). Virus stock for RNA extraction was prepared by a single passage on Vero cell monolayers (ATCC CCL-81).

RNA was extracted from clarified Vero cell supernatant using the High Pure RNA Isolation kit (Roche), as per the manufacturer’s instructions, with some modification. Briefly, clarified supernatant was filtered and concentrated by ultracentrifugation (4,000 × *g*, 20 min) in Millipore Amicon Ultra-15 centrifugal units (Merck). Concentrated filtered virus supernatant (200 μl) was then emulsified in 400 μl of Roche lysis buffer. RNA extraction then proceeded as per the standard protocol. RNA was quantitated and assessed for quality using the NanoDrop 2000 spectrophotometer (Thermo Fisher Scientific) and subsequently diluted to within working range of more sensitive analysis with the Qubit 2.0 fluorometer (Qubit RNA HS assay kit; Invitrogen) in order to standardize RNA for Illumina library preparation.

### Whole-genome sequencing.

The TruSeq Stranded mRNA Library Prep kit (96 reactions; Illumina) was used to generate library preparations, as per the manufacturers’ instructions, modified to exclude the poly(A)-containing mRNA purification steps. RNA was reverse transcribed using SuperScript III reverse transcriptase (Invitrogen) and random hexanucleotide primers (Illumina). Resulting libraries were validated using the Agilent 1000 DNA kit (Integrated Sciences). Validated libraries were then normalized and pooled, including a Phi-X control v3 (Illumina) to a final concentration of 12.5 pM. The final denatured pool was loaded onto a MiSeq reagent kit v2, 300 cycles (Illumina), before sequencing on a MiSeq. Reads were demultiplexed and assessed for quality using FastQC v0.11 (45). Full coding consensus sequences were assembled using CLC Genomics Workbench v7.5 (Qiagen) and Geneious v11.1.

### Phylogenetic and variant analysis.

Complete coding regions were aligned using MAFFT 7.338 (46), as implemented in Geneious v11.1, and then manually trimmed to remove 5′ and 3′ noncoding regions for phylogenetic analyses. JModelTest v2.1 (47) was employed to determine the best fit nucleotide substitution model (GTR + Г_4_ + I) for the WGS data set for subsequent phylogenetic reconstructions based on the Bayesian information criterion (BIC).

Maximum likelihood (ML) phylogenies were reconstructed using RAxML v8.2 (48) with a general time reversible nucleotide substitution model with gamma rate heterogeneity and proportion of invariable sites (GTR + Г_4_ + I). Branch support was estimated using 1,000 maximum likelihood bootstrap replicates. ML trees were visualized and edited within FigTree v1.4 (49) in order to characterize distinct genetic groupings of RRV.

### Nucleotide substitution rate, divergence times, and geographical structure of RRV.

To estimate the nucleotide substitution rate and the divergence times of RRV genotypes and sublineages, Bayesian Markov Chain Monte Carlo (MCMC) analysis was conducted within the BEAST v1.8 (50) package to generate a median clade credibility (MCC) tree. Prior to Bayesian analysis, TempEst v1.5 (51) was employed to assess the temporal signal of the data set prior to analysis. A high degree of clock-like behavior was observed (*R*^2^ = 0.879) in root-to-tip regression analysis, suggesting temporal signal and appropriateness of the data set for estimation of temporal parameters. The GTR + Г_4_ + I nucleotide substitution model was applied to the data set, under an uncorrelated log normal (UCLN) relaxed molecular clock with a constant-size demographic model. Default prior settings were used for all parameters. Three independent MCMC chains were run and subsequently assessed for convergence (ESS values of >200 for all parameters) within Tracer v1.7 (52). MCC trees were inferred with an appropriate 10% burn-in within TreeAnnotator v1.8 before visualization and editing within FigTree v1.4. To define the geographical structure of phylogenetic groups, isolates were coded as belonging to one of five geographical categories: north WA, south WA, central WA, Pacific Island countries and territories (PICTs), or Australia excluding WA.

Nucleotide substitution rates and the time to most recent common ancestor (tMRCA) were estimated for the complete data set, with statistical error reported as the 95% highest probability density (95% HPD).

Changes in relative genetic diversity of RRV were estimated using a flexible skyline coalescent demographic model (53).

### Selection pressure detection.

Selection pressures acting along the codon regions were determined with the maximum likelihood alignment of the 106 taxa data set to identify sites under negative (purifying) or positive (diversifying) selection pressure. The two separate ORFs were analyzed independently to exclude the junction noncoding region. Four methods of selection detection were employed using HyPhy through the DataMonkey webserver (www.datamonkey.org/), specifically, fixed-effect likelihood (FEL) (54), mixed-effects model of evolution (MEME) (55), fast, unconstrained Bayesian approximation (FUBAR) (56), and single-likelihood ancestor counting (SLAC) (54). Codon sites under negative or positive selection were confirmed by at least two methods with a *P* value of <0.05 for FEL and MEME, a *P* value of <0.1 for SLAC, and a posterior probability of over 90% in FUBAR.

### Data availability.

All sequences derived for this study were deposited in GenBank and assigned accession numbers MN038196 to MN038289. The accession numbers for all isolates utilized in this investigation, including those downloaded from GenBank, are provided in Table S1.

## Supplementary Material

Supplemental file 1
