# Supplementary material for: Genome-Scale Phylogeny and Evolutionary Analysis of Ross River Virus Reveals Periodic Sweeps of Lineage Dominance in Western Australia, 1977–2014
Source: J Virol. 2020 Jan 6;94(2):e01234-19. doi: 10.1128/JVI.01234-19 (PMC6955267; doi:10.1128/JVI.01234-19)
Supplement: Supplemental file 1 [file JVI.01234-19-s0001.pdf]

Genome-scale Phylogeny and Evolutionary Analysis of Ross River virus Reveals Periodic Sweeps of Lineage Dominance in Western Australia, 1977 – 2014.

Supplementary Table 1: Details of isolates sequenced or accessed from NCBI for the purposes of this study.

Isolate Name	Location of Collection	Collection Date	Source Species	Accession Number
218072	PICTs, Cook Islands	1980	*Homo sapiens*	MN038196
218081	PICTs, Cook Islands	1980	*H. sapiens*	MN038197
218100	PICTs, Cook Islands	1980	*H. sapiens*	MN038198
218397	PICTs, Cook Islands	1980	*H. sapiens*	MN038199
AN205	Western Australia, Argyle Mine	18/03/1982	*Aedes normanensis*	MN038200
AN572.1	Western Australia, Kununurra	13/02/1984	*Ae. normanensis*	MN038201
AN572.2	Western Australia, Kununurra	13/02/1984	*Ae. normanensis*	MN038202
DC266	Western Australia, Mandurah	22/06/1994	*Ae. camptorhynchus*	MN038203
DC7053	Western Australia, Mandurah	27/12/2005	*Ae. camptorhynchus*	MN038204
DC17878	Western Australia, Mandurah	13/10/1998	*Ae. camptorhynchus*	MN038205
DC29695	Western Australia, Mandurah	30/10/2000	*Ae. camptorhynchus*	MN038206

DC30176	Western Australia, Mandurah	29/11/2000	*Ae. vigilax*	MN038207
DC30218	Western Australia, Rockingham	12/12/2000	*Ae. vigilax*	MN038208
DC36025	Western Australia, Mandurah	16/09/2003	*Ae. camptorhynchus*	MN038209
DC36486	Western Australia, Mandurah	11/11/2003	*Ae. camptorhynchus*	MN038210
DC36571	Western Australia, Mandurah	11/11/2003	*Ae. camptorhynchus*	MN038211
DC36664	Western Australia, Mandurah	25/11/2003	*Anopheles annulipes*	MN038212
DC40243	Western Australia, Mandurah	6/12/2005	*Ae. camptorhynchus*	MN038213
DC55607	Western Australia, Waroona	4/12/2012	*Ae. camptorhynchus*	MN038214
DC59627	Western Australia, Mandurah	30/09/2014	*Ae. camptorhynchus*	MN038215
F9073	PICTs, Fiji	1979	*H. sapiens*	MN038216
K1008	Western Australia, Kununurra	2/03/1986	*Ae. normanensis*	MN038217
K1198	Western Australia, Parry's Creek	7/02/1984	*Culex annulirostris*	MN038218
K1503	Western Australia, Parry's Creek	26/01/1984	*Cx. annulirostris*	MN038219
K2505	Western Australia, Kununurra	5/03/1984	*Cx. annulirostris*	MN038220
K3011	Western Australia, Nullagine	16/02/1990	*Ae. pseudonormanensis*	MN038221
K22383	Western Australia, Willare	29/03/1996	*Ae. normanensis*	MN038222

K44441	Western Australia, Billiluna	29/03/2001	*Ae. normanensis*	MN038223
K47755	Western Australia, Geikie Gorge	15/4/2001	*Ae. normanensis*	MN083224
K50081	Western Australia, Roebuck Plain	11/04/2002	*Cx. annulirostris*	MN038225
K50610	Western Australia, Billiluna	15/03/2003	*Ae. normanensis*	MN038226
K51670	Western Australia, Wyndham	19/03/2003	*Cx. annulirostris*	MN038227
K61297	Western Australia, Kununurra	23/03/2006	*Cx. annulirostris*	MN038228
K65195	Western Australia, Derby	20/04/2007	*Ae. vigilax*	MN038229
K67847	Western Australia, Roebuck Plain	1/04/2008	*Cx. annulirostris*	MN038230
K70883	Western Australia, Halls Creek	23/03/2010	*Cx. annulirostris*	MN038231
K70905	Western Australia, Halls Creek	23/03/2010	*Ae. tremulus*	MN038232
K76352	Western Australia, Coconut Wells	29/03/2012	*Cx. annulirostris*	MN038233
K78118	Western Australia, Kununurra	6/04/2012	*Mansonia uniformis*	MN038234
K79390	Western Australia, Wyndham	10/04/2012	*Cx. annulirostris*	MN038235
K80535	Western Australia, Broome	20/03/2013	*Cx. sitiens*	MN038236
K80776	Western Australia, Derby	21/03/2013	*Cx. annulirostris*	MN038237
NCO358	Western Australia, Mullewa	18/05/2000	*Ae. bancroftianus*	MN038238

P1159	Western Australia, Whim Creek	11/03/1994	*Ae. pseudonormanensis*	MN038239
P1165	Western Australia, Balla Balla	11/03/1994	*Ae. eidsvoldensis*	MN038240
P1170	Western Australia, Whim Creek	11/03/1994	*Cx. annulirostris*	MN038241
P1373	Western Australia, Pardoo	14/03/1994	*An. amictus*	MN038242
P5131	Western Australia, Newman	7/03/1997	*Ae. normanensis*	MN038243
P6179	Western Australia, Exmouth	27/04/1999	*Ae. vigilax*	MN038244
P6265	Western Australia, Exmouth	27/04/1999	*Cx. annulirostris*	MN038245
P6273	Western Australia, Exmouth	14/04/1999	*Ae. vigilax*	MN038246
P6298	Western Australia, Exmouth	14/04/1999	*Ae. vigilax*	MN038247
P41453	PICTs, Fiji	1979	*H. sapiens*	MN038248
P41472	PICTs, American Samoa	1979	*H. sapiens*	MN038249
P41971	PICTs, Cook Islands	1980	*H. sapiens*	MN038250
P42115	PICTs, Cook Islands	1980	*H. sapiens*	MN038251
P42134	PICTs, Cook Islands	1980	*H. sapiens*	MN038252
P42161	PICTs, Cook Islands	1980	*H. sapiens*	MN038253
P42213	PICTs, Cook Islands	1980	*H. sapiens*	MN038254

P42273	PICTs, Cook Islands	1980	*H. sapiens*	MN038255
SE1168	Western Australia, Kalgoorlie	3/05/2013	*Ae. bancroftianus*	MN038256
SHLS735	Western Australia, Pinjarra	6/01/1989	*H. sapiens*	MN038257
SHLS2173	Western Australia, Bunbury	11/01/1989	*H. sapiens*	MN038258
SW876	Western Australia, Waroona	19/08/1987	*Ae. camptorhynchus*	MN038259
SW2089	Western Australia, Mandurah	9/11/1988	*An. annulipes sensu lato (s.l.)*	MN038260
SW2191	Western Australia, Harvey	9/11/1988	*Ae. camptorhynchus*	MN038261
SW3181	Western Australia, Murray	21/09/1989	*Ae. camptorhynchus*	MN038262
SW11747	Western Australia, Mandurah	24/10/1990	*Ae. camptorhynchus*	MN038263
SW12358	Western Australia, Harvey	5/12/1990	*Ae. camptorhynchus*	MN038264
SW20275	Western Australia, Busselton	17/10/1991	*Ae. camptorhynchus*	MN038265
SW20733	Western Australia, Cockburn	20/02/1992	*Coquillettidia species near linealis*	MN038266
SW23448	Western Australia, Mount Magnet	1/04/1992	*Ae. sagax*	MN038267
SW23656	Western Australia, Exmouth	22/05/1992	*Ae. vigilax*	MN038268
SW24015	Western Australia, Marble Bar	16/05/1992	*Ae. tremulus*	MN038269

SW24336	Western Australia, Exmouth	17/06/1992	*Ae. E.N. Marks' species No. 85*	MN038270
SW29862	Western Australia, Onslow	13/06/1992	*Ae. E.N. Marks' species No. 85*	MN038271
SW38788	Western Australia, Busselton	5/12/1994	*Ae. camptorhynchus*	MN038272
SW42256	Western Australia, Dardanup	15/01/1996	*Ae. camptorhynchus*	MN038273
SW58968	Western Australia, Bunbury	4/10/1999	*Ae. clelandi*	MN038274
SW64247	Western Australia, Capel	11/10/2000	*Ae. camptorhynchus*	MN038275
SW71959	Western Australia, Capel	14/10/2003	*Ae. camptorhynchus*	MN038276
SW71961	Western Australia, Capel	14/10/2003	*Ae. camptorhynchus*	MN038277
SW72209	Western Australia, Busselton	30/10/2003	*Ae. camptorhynchus*	MN038278
SW72718	Western Australia, Busselton	9/12/2003	*Ae. camptorhynchus*	MN038279
SW72780	Western Australia, Busselton	22/12/2003	*Ae. alboannulatus*	MN038280
SW72961	Western Australia, Dardanup	4/03/2004	*Ae. alboannulatus*	MN038281
SW74249	Western Australia, Harvey	16/11/2004	*Ae. camptorhynchus*	MN038282
SW83959	Western Australia, Bunbury	28/10/2008	*Cx. globocoxitus*	MN038283
SW94735	Western Australia, Harvey	2/01/2013	*Ae. camptorhynchus*	MN038284
SW97414	Western Australia, Capel	26/11/2013	*Ae. camptorhynchus*	MN038285

SW99359	Western Australia, Harvey	28/10/2014	*Ae. camptorhynchus*	MN038286
SWDN	Western Australia, Australind	13/12/1991	*H. sapiens*	MN038287
SWSM	Western Australia, Nannup	3/05/1992	*H. sapiens*	MN038288
WK20	Western Australia, Derby	1/04/1977	*Cx. annulirostris*	MN038289
DC5692	Western Australia, Peel region	1996	*Ae. camptorhynchus*	HM235643
RRV_TT	Queensland	2014	*H. sapiens*	KY302801
MIDI13	Queensland	2016	*H. sapiens*	MH987779
MIDI32	Queensland	2017	*H. sapiens*	MH987780
MIDI86	Queensland	2018	*Verrallina funerea*	MH987781
QML-1	Queensland	2004	*H. sapiens*	GQ433354
2982	Queensland	1965	*Microeca fasinans*	GQ433355
3078	Queensland	1965	*Poephila personata*	GQ433356
8691	Queensland	1965	*Macropus agilis*	GQ433357
9057	Queensland	1968	*M. agilis*	GQ433358
T48	Queensland, Townsville	1959	*Ae. vigilax*	GQ433359

2975	Queensland	1965	*Grallina cyanoleuca*	GQ433360

Supplementary Table 2: Unique amino acid substitutions, and their locations within the genome, that are specific to distinct genotypes or sub-lineages of RRV. The nature of the mutation, that is, whether it is a conservative or non-conservative mutation, is noted.

Gene	Amino Acid Transition and Location within gene	Defining Genotype/Sub-lineage	Substitution type
nsP1	T56A	G2, except DC5692	Non-Conservative
	T116A	G4A	Non-Conservative
	L224I	G2	Conservative
	C416F	G2B	Conservative
	S424N	G2B	Conservative
	E501D	G4	Conservative
nsP2	T31A	G1	Non-Conservative
	K34R	G2A	Non-Conservative
	T175A	G2	Non-Conservative
	N219T	G3	Non-Conservative
	S406T	G2B	Conservative
nsP3	I192V	G2B	Conservative
	I284V	Only PICTS G3	Conservative
	T422I	G4	Non-Conservative
	T427K	G1	Non-Conservative
	A431T	G2	Non-Conservative
	T456I	G4B, except RRV_TT	Non- Conservative
	E494K	G1	Non-Conservative
	E516A	G2A	Non-Conservative
	P518S	G1	Non-Conservative
nsP4	Q49L	G4	Non-Conservative
	T89A	G2	Non-Conservative
	V99A	G2	Conservative
	A359V	G2	Conservative
	E399D	G2	Conservative
	I438V	G1	Conservative
	K514N	G2	Non-Conservative
6k*	F46I	G4B	Conservative
	F46L	G2A	Conservative
	A58V	G4	Conservative
Capsid*	M33V	G2A	Conservative
	T35A	G4	Non-Conservative
	K73Q	G2	Non-Conservative

E1*	L120S	G1	Non-Conservative
	V426L	G4	Conservative
E2*	I68M	G2	Conservative
	E302V	G2	Conservative

* Structural proteins

Supplementary Figure 1: Maximum likelihood phylogeny (RAxML) reconstructed with the 106 whole genome dataset, as well as all geographically characterised published E2 sequences of RRV. Virus nomenclature includes the strain name, location of collection, species of origin and the year of sampling. GenBank accession numbers are provided for sequences derived from NCBI. Taxa are coloured based on their geographical origin: Red, Western Australia; Purple, Queensland; Green, New South Wales; Blue, Northern Territory; Indigo, Victoria; Pink, Tasmania; Orange, the Pacific Island Countries and Territories . Bootstrap support values over 70% are presented above nodes.

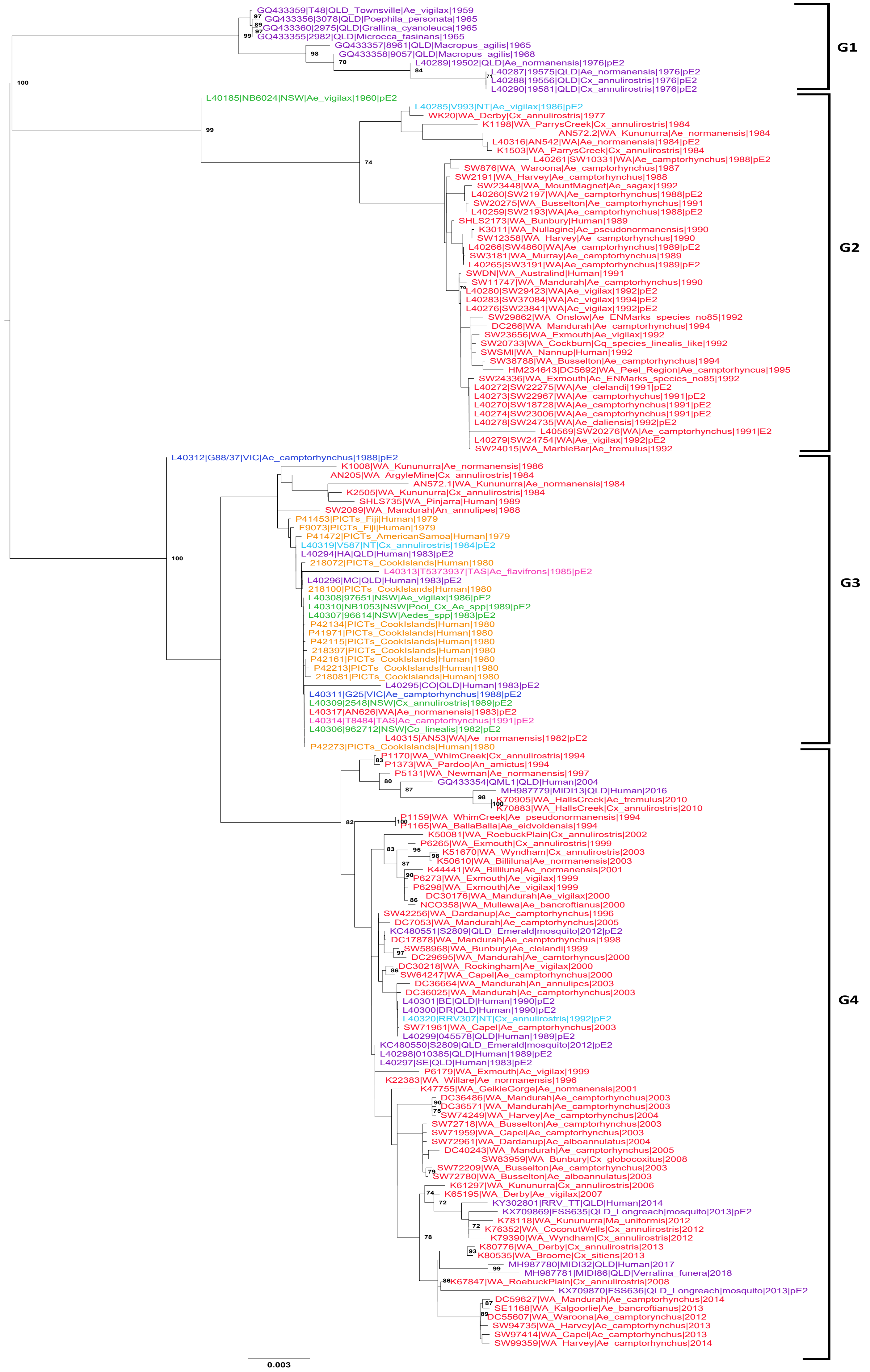

G1
G2
G3
G4
GQ433359|T48|QLD_Townsville|Ae_vigilax|1959
GQ433356|3078|QLD|Poephila_personata|1965
GQ433360|2975|QLD|Grallina_cyanoleuca|1965
GQ433355|2982|QLD|Microeca_fasinans|1965
GQ433357|8961|QLD|Macropus_agilis|1965
GQ433358|9057|QLD|Macropus_agilis|1968
L40289|19502|QLD|Ae_normanensis|1976|pE2
L40287|19575|QLD|Ae_normanensis|1976|pE2
L40288|19556|QLD|Cx_annulirostris|1976|pE2
L40290|19581|QLD|Cx_annulirostris|1976|pE2
L40185|NB6024|NSW|Ae_vigilax|1960|pE2
L40285|V993|NT|Ae_vigilax|1986|pE2
WK20|WA_Derby|Cx_annulirostris|1977
K1198|WA_ParrysCreek|Cx_annulirostris|1984
AN572.2|WA_Kununurra|Ae_normanensis|1984
L40316|AN542|WA|Ae_normanensis|1984|pE2
K1503|WA_ParrysCreek|Cx_annulirostris|1984
L40261|SW10331|WA|Ae_camptorhynchus|1988|pE2
SW876|WA_Waroona|Ae_camptorhynchus|1987
SW2191|WA_Harvey|Ae_camptorhynchus|1988
SW23448|WA_MountMagnet|Ae_sagax|1992
L40260|SW2197|WA|Ae_camptorhynchus|1988|pE2
SW20275|WA_Busselton|Ae_camptorhynchus|1991
L40259|SW2193|WA|Ae_camptorhynchus|1988|pE2
SHLS2173|WA_Bunbury|Human|1989
K3011|WA_Nullagine|Ae_pseudonormanensis|1990
SW12358|WA_Harvey|Ae_camptorhynchus|1990
L40266|SW4860|WA|Ae_camptorhynchus|1989|pE2
SW3181|WA_Murray|Ae_camptorhynchus|1989
L40265|SW3191|WA|Ae_camptorhynchus|1989|pE2
SWDN|WA_Australind|Human|1991
SW11747|WA_Mandurah|Ae_camptorhynchus|1990
L40280|SW29423|WA|Ae_vigilax|1992|pE2
L40283|SW37084|WA|Ae_vigilax|1994|pE2
L40276|SW23841|WA|Ae_vigilax|1992|pE2
SW29862|WA_Onslow|Ae_ENMarks_species_no85|1992
DC266|WA_Mandurah|Ae_camptorhynchus|1994
SW23656|WA_Exmouth|Ae_vigilax|1992
SW20733|WA_Cockburn|Cq_species_linealis_like|1992
SWSMI|WA_Nannup|Human|1992
SW38788|WA_Busselton|Ae_camptorhynchus|1994
HM234643|DC5692|WA_Peel_Region|Ae_camptorhyncus|1995
SW24336|WA_Exmouth|Ae_ENMarks_species_no85|1992
L40272|SW22275|WA|Ae_clelandi|1991|pE2
L40273|SW22967|WA|Ae_camptorhychus|1991|pE2
L40270|SW18728|WA|Ae_camptorhynchus|1991|pE2
L40274|SW23006|WA|Ae_camptorhynchus|1991|pE2
L40278|SW24735|WA|Ae_daliensis|1992|pE2
L40569|SW20276|WA|Ae_camptorhynchus|1991|E2
L40279|SW24754|WA|Ae_vigilax|1992|pE2
SW24015|WA_MarbleBar|Ae_tremulus|1992
L40312|G88/37|VIC|Ae_camptorhynchus|1988|pE2
K1008|WA_Kununurra|Ae_normanensis|1986
AN205|WA_ArgyleMine|Cx_annulirostris|1984
AN572.1|WA_Kununurra|Ae_normanensis|1984
K2505|WA_Kununurra|Cx_annulirostris|1984
SHLS735|WA_Pinjarra|Human|1989
SW2089|WA_Mandurah|An_annulipes|1988
P41453|PICTs_Fiji|Human|1979
F9073|PICTs_Fiji|Human|1979
P41472|PICTs_AmericanSamoa|Human|1979
L40319|V587|NT|Cx_annulirostris|1984|pE2
L40294|HA|QLD|Human|1983|pE2
218072|PICTs_CookIslands|Human|1980
L40313|T5373937|TAS|Ae_flavifrons|1985|pE2
L40296|MC|QLD|Human|1983|pE2
218100|PICTs_CookIslands|Human|1980
L40308|97651|NSW|Ae_vigilax|1986|pE2
L40310|NB1053|NSW|Pool_Cx_Ae_spp|1989|pE2
L40307|96614|NSW|Aedes_spp|1983|pE2
P42134|PICTs_CookIslands|Human|1980
P41971|PICTs_CookIslands|Human|1980
P42115|PICTs_CookIslands|Human|1980
218397|PICTs_CookIslands|Human|1980
P42161|PICTs_CookIslands|Human|1980
P42213|PICTs_CookIslands|Human|1980
218081|PICTs_CookIslands|Human|1980
L40295|CO|QLD|Human|1983|pE2
L40311|G25|VIC|Ae_camptorhynchus|1988|pE2
L40309|2548|NSW|Cx_annulirostris|1989|pE2
L40317|AN626|WA|Ae_normanensis|1983|pE2
L40314|T8484|TAS|Ae_camptorhynchus|1991|pE2
L40306|962712|NSW|Co_linealis|1982|pE2
L40315|AN53|WA|Ae_normanensis|1982|pE2
P42273|PICTs_CookIslands|Human|1980
P1170|WA_WhimCreek|Cx_annulirostris|1994
P1373|WA_Pardoo|An_amictus|1994
P5131|WA_Newman|Ae_normanensis|1997
GQ433354|QML1|QLD|Human|2004
MH987779|MIDI13|QLD|Human|2016
K70905|WA_HallsCreek|Ae_tremulus|2010
K70883|WA_HallsCreek|Cx_annulirostris|2010
P1159|WA_WhimCreek|Ae_pseudonormanensis|1994
P1165|WA_BallaBalla|Ae_eidvoldensis|1994
K50081|WA_RoebuckPlain|Cx_annulirostris|2002
P6265|WA_Exmouth|Cx_annulirostris|1999
K51670|WA_Wyndham|Cx_annulirostris|2003
K50610|WA_Billiluna|Ae_normanensis|2003
K44441|WA_Billiluna|Ae_normanensis|2001
P6273|WA_Exmouth|Ae_vigilax|1999
P6298|WA_Exmouth|Ae_vigilax|1999
DC30176|WA_Mandurah|Ae_vigilax|2000
NCO358|WA_Mullewa|Ae_bancroftianus|2000
SW42256|WA_Dardanup|Ae_camptorhynchus|1996
DC7053|WA_Mandurah|Ae_camptorhynchus|2005
KC480551|S2809|QLD_Emerald|mosquito|2012|pE2
DC17878|WA_Mandurah|Ae_camptorhynchus|1998
SW58968|WA_Bunbury|Ae_clelandi|1999
DC29695|WA_Mandurah|Ae_camtorhyncus|2000
DC30218|WA_Rockingham|Ae_vigilax|2000
SW64247|WA_Capel|Ae_camptorhynchus|2000
DC36664|WA_Mandurah|An_annulipes|2003
DC36025|WA_Mandurah|Ae_camptorhynchus|2003
L40301|BE|QLD|Human|1990|pE2
L40300|DR|QLD|Human|1990|pE2
L40320|RRV307|NT|Cx_annulirostris|1992|pE2
SW71961|WA_Capel|Ae_camptorhynchus|2003
L40299|045578|QLD|Human|1989|pE2
KC480550|S2809|QLD_Emerald|mosquito|2012|pE2
L40298|010385|QLD|Human|1989|pE2
L40297|SE|QLD|Human|1983|pE2
P6179|WA_Exmouth|Ae_vigilax|1999
K22383|WA_Willare|Ae_normanensis|1996
K47755|WA_GeikieGorge|Ae_normanensis|2001
DC36486|WA_Mandurah|Ae_camptorhynchus|2003
DC36571|WA_Mandurah|Ae_camptorhynchus|2003
SW74249|WA_Harvey|Ae_camptorhynchus|2004
SW72718|WA_Busselton|Ae_camptorhynchus|2003
SW71959|WA_Capel|Ae_camptorhynchus|2003
SW72961|WA_Dardanup|Ae_alboannulatus|2004
DC40243|WA_Mandurah|Ae_camptorhynchus|2005
SW83959|WA_Bunbury|Cx_globocoxitus|2008
SW72209|WA_Busselton|Ae_camptorhynchus|2003
SW72780|WA_Busselton|Ae_alboannulatus|2003
K61297|WA_Kununurra|Cx_annulirostris|2006
K65195|WA_Derby|Ae_vigilax|2007
KY302801|RRV_TT|QLD|Human|2014
KX709869|FSS635|QLD_Longreach|mosquito|2013|pE2
K78118|WA_Kununurra|Ma_uniformis|2012
K76352|WA_CoconutWells|Cx_annulirostris|2012
K79390|WA_Wyndham|Cx_annulirostris|2012
K80776|WA_Derby|Cx_annulirostris|2013
K80535|WA_Broome|Cx_sitiens|2013
MH987780|MIDI32|QLD|Human|2017
MH987781|MIDI86|QLD|Verralina_funera|2018
K67847|WA_RoebuckPlain|Cx_annulirostris|2008
KX709870|FSS636|QLD_Longreach|mosquito|2013|pE2
DC59627|WA_Mandurah|Ae_camptorhynchus|2014
SE1168|WA_Kalgoorlie|Ae_bancroftianus|2013
DC55607|WA_Waroona|Ae_camptoryanchus|2012
SW94735|WA_Harvey|Ae_camptorhynchus|2013
SW97414|WA_Capel|Ae_camptorhynchus|2013
SW99359|WA_Harvey|Ae_camptorhynchus|2014
0.003
